# Grip and manipulation forces are controlled independently in a coupled bimanual task

**DOI:** 10.1186/s12984-025-01600-4

**Published:** 2025-03-11

**Authors:** Clara Günter, Niklas Heimburger, David W. Franklin, Raz Leib

**Affiliations:** 1 Neuromuscular Diagnostics, School of Medicine and Health, Technical University of Munich, Munich, Germany; 2 Munich Institute of Robotics and Machine Intelligence (MIRMI), Technical University of Munich, Munich, Germany; 3 Munich Data Science Institute (MDSI), Technical University of Munich, Munich, Germany

**Keywords:** Bimanual manipulation, Grip force, Force control, Object manipulation

## Abstract

**Background:**

Grasping and manipulating objects requires humans to adapt both grip and manipulation forces. When handling an object with both hands, the additional degrees of freedom introduce more levels to the redundancy of the object manipulation since we can distribute the contribution of the grip and manipulation forces between hands.

**Methods:**

In this study, we investigated the forces produced by both hands during coupled bimanual manipulation of a needle object in a virtual environment. The task objective was to puncture a virtual tissue, modeled as a linear spring, and stop immediately after, with the hands arranged in front and back positions in the movement direction.

**Results:**

We show that during tissue interaction, grip forces are modulated consistently between front and back hands across participants, but manipulation forces are not. That is, the back hand consistently produced excessive grip force compared to the front hand regardless of hand configuration, while manipulation force distribution between the two hands was variable. After the tissue puncture, we again observed consistent grip force behavior during the reactive response to the force drop following the puncture. The grip force signal exhibited a consistent temporal profile in both the front and back hands with amplitude modulation according to the tissue stiffness in the front hand.

**Conclusions:**

Overall, our results support the idea of distinct control mechanisms for grip and manipulation forces which rely on hand position rather than hand dominance.

## Introduction

To efficiently and dexterously manipulate an object, it is necessary to generate both the appropriate grip forces to prevent the object from slipping from our grasp and the needed manipulation forces to control the object’s trajectory. Both grip and manipulation forces must be tuned to the object’s mechanical properties, such as mass or stiffness [1], and to the forces originating in the environment, such as viscoelastic or gravitational forces [2, 3]. While planning and adjusting our manipulation forces during interaction with an object is a challenging task when using a single hand, bimanual object manipulation increases the difficulty level further. One issue that is emphasized during bimanual object manipulation is the additional redundancy we need to solve [4, 5]. That is, in bimanual manipulation, we can choose and switch the control role of each hand. For example, when inserting a straw into the plastic lid of a takeaway cup, one hand needs to compensate for a sudden drop in stiffness when the straw has pierced the lid. At the same time, the other hand has to hold the cup in place. Although most individuals prefer using one hand for the pushing role and one hand for the stabilizing role [6–9], we can complete this task in arbitrary hand configurations. How the sensorimotor system solves this redundancy is still unknown.

While redundancy can refer to multiple levels of control in the motor system, here, we define it as the hand effort distribution in a mechanically coupled task where both hands have a common goal. That is, there is an infinite number of effort distributions between the hands that can lead to the successful completion of a task. As with the example of inserting a straw into a plastic lid, a possible way to solve this redundancy is to produce asymmetric movements with a specialised role assigned to each hand. Most research comparing the roles of the left and right hands have used point-to-point reaching movements [6, 9, 10]. Since these studies focused on movement in free space, they have examined how the redundancy is solved in terms of movement kinematics. However, examining more complex tasks, especially ones that require force production and adaptation to external forces, can serve as a different approach to unveil manipulation strategies that can solve the bimanual redundancy problem [11].

When manipulating an object, we need to generate manipulation forces that will move the object in space (trajectory control) and grip forces to firmly hold the object. We apply grip forces on the object’s surfaces to generate frictional forces that resist external forces so the object will not slip from our fingers [12, 13]. In many scenarios, the sensorimotor system tries to predict the nature of the coming environmental forces, which can be gravitational forces [1], inertia due to self-motion [3, 14, 15], or general forces generated by the environment [2, 16], and modulates the grip forces accordingly. For example, before we lift an object, we apply grip forces that consider the object’s weight and friction, how fast we will lift it, and whether it is loosely attached to the ground. Thus, due to its predictive nature, the nature and modulation of applied grip forces can indicate different control strategies during object manipulation [17] and, moreover, during bimanual object manipulation.

Although usually coupled, manipulation and grip forces could act independently from each other. It has been suggested that the two forces are controlled [18–20] and planned [21] individually. For example, removing force feedback [22], elevation of tactile feedback (skin-stretch) [23], or increased uncertainty in the environment [24] led to elevated baseline grip forces, while the manipulation forces were unaffected, indicating distinct control mechanisms. While some work extended the results from unimanual to bimanual object manipulation in uncoupled scenarios [25–27], coupled scenarios received less attention. A recent study by Takagi and Kashino showed changeable role distribution between the dominant and non-dominant hands, which depends on the arms’ posture and movement target, with a general increase in endpoint impedance (measured as grasping forces) when moving against large diverting forces [28]. However, whether such role distribution between hands in terms of force production and especially precision grip force behavior exists in coupled bimanual tasks is still an open question.

To fill this gap, we designed an experiment where participants bimanually inserted a needle into a simulated tissue, which generated environmental forces, with their hands placed behind one another in the manipulation direction. By modifying the hand configuration and the simulated tissue stiffness, we generated different requirements for interaction and grip forces to examine different strategies that might be used. Since grip force control appears to be based more on unconscious processes [29] while manipulation force generation can result from conscious, explicit strategy [30], we hypothesized that grip force strategy will remain consistent across experimental conditions, whereas manipulation forces would exhibit more variable solutions.

## Methods

We examined grip force and manipulation force control during bimanual manipulation of a simulated needle object in a needle insertion task.

**Fig. 1:**
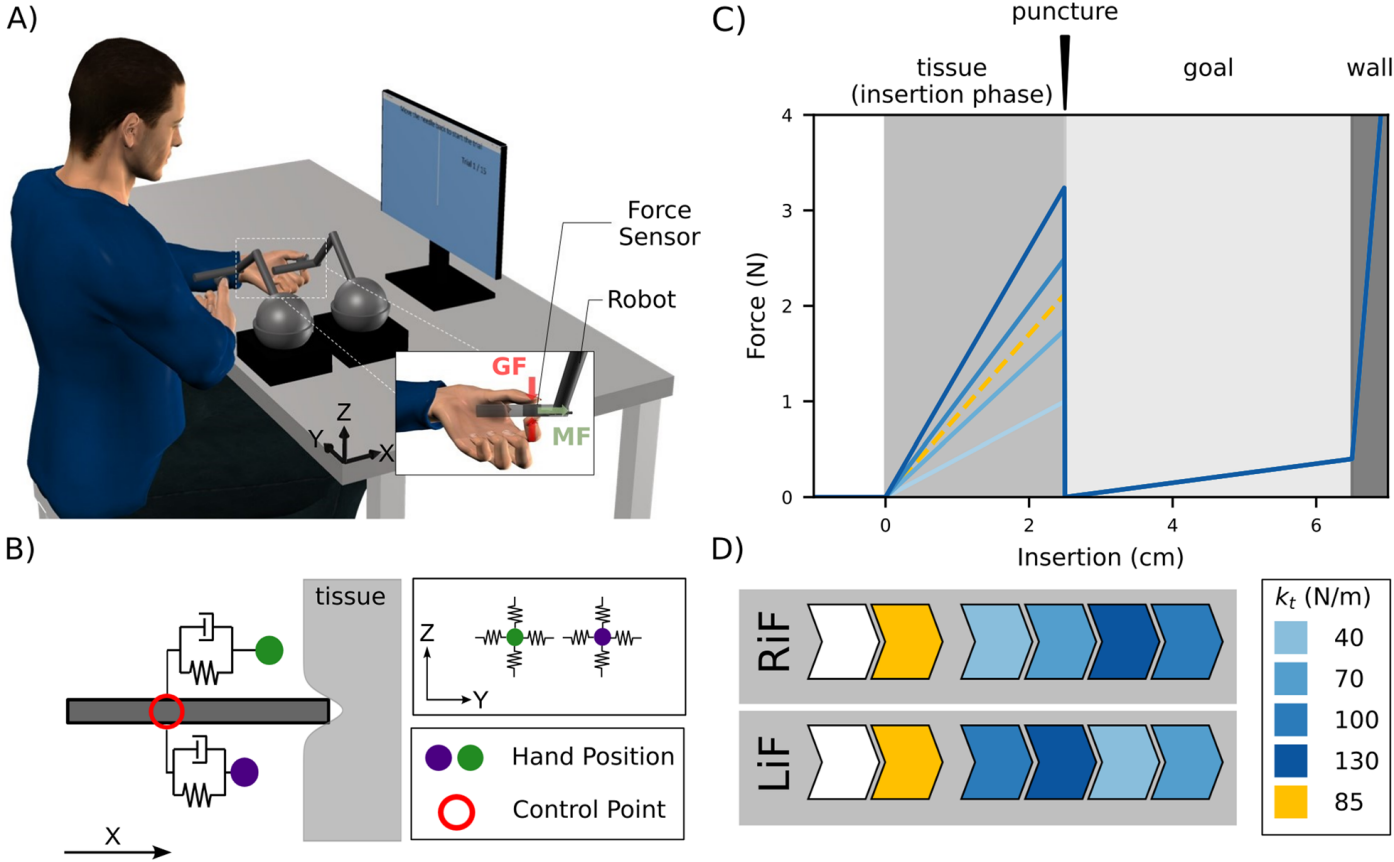
Experimental setup. **a** The participant holds two grip force sensors connected to haptic robots while observing a screen that was placed in front of them, applying manipulation force (MF) and grip force (GF) to the robot and sensors, respectively. **b** The experiment dynamics. Each robot (hand position) controlled by the participant, is connected to the needle object (control point) via a spring-damper system in the X-direction. Movement in the YZ-plane was limited by a force channel. The tissue in the experiment was represented by one of four springs. **c** The four spring constants and position-dependent forces outside tissue (white region), in the tissue (medium grey region), in the goal (light grey region) and in the wall (dark grey region) zones. Participants were instructed to move through the tissue zone and stop as quickly as possible after penetrating it, reducing the maximum insertion into the goal zone. The wall prevented participants from moving outside the robotic range of motion. **d** An example experimental protocol. Familiarization included one block of 15 trials without tissue forces (white) and one block with the average of tissue forces encountered in the experiment (orange), before they experienced each of the four tissue stiffnesses (blue conditions). The order of Right in Front (RiF) and Left in Front (LiF) and of $k_t$ was pseudo-randomized for each participant

### Participants

Fourteen right-handed (median handedness score: 95, assessed using the Edinburgh Inventory [31]) participants (eight males, six females, aged $26\pm 5$ years) participated in the experiment. Before the experiment, participants were introduced to and familiarized with the haptic devices and provided written informed consent. All participants were neurologically healthy and naïve to the purpose of the study. The institutional ethics committee at TUM approved the study.

### Experimental setup

Participants sat in front of a screen and grasped with each hand one of two ATI Nano25-E transducers, each attached to a haptic robot interface (Phantom Touch; 3D SYSTEMS) with a 3D-printed adapter and covered with 800P sandpaper (see Fig. 1a). Using their index finger and thumb, participants grasped each sensor. The front hand was held in a neutral position with the thumb on top and the rear hand in a pronated position with the thumb towards their midline, while sitting at approximately $45^\circ$, facing the robots with the rear shoulder in line with the movement axis. The task was to insert a virtual needle through a tissue and into a target area using both hands. Participants were instructed to hold both sensors perpendicular to the movement direction. Between trials, the needle object was visible on the screen; during trials, we removed all visual feedback from the screen. The experiment was conducted in a virtual reality environment using CHAI3d [32] to render forces and visual representations.

### Virtual needle-tissue model

The virtual needle was simulated using a mechanical system in which participants generated forces on the needle object at the same control point for both hands (see Fig. 1b). That is, we simulated the virtual needle as a point mass and attached the two control points of the robots to this point without any virtual biases, although the robots, and therefore the hands, were physically at different locations (see Fig. 1a). Participants could manipulate the needle and received force feedback based on the position of the needle object within the environment consisting of four regions: free space, tissue, goal, and blocking wall (see Fig. 1b). Each region was simulated as a spring, with different stiffness values, and the forces in each region were calculated according to the difference in needle position from the boundary point of the region. The first area was free space in which participants did not experience any forces. The following area was the tissue in which we used one out of four possible spring values ($k_t$, see Table 1) and the start boundary point was set at $x_n$ = 0 cm. In the goal region, that is, after the puncture of the tissue (see Fig. 1c), $k_t$ was replaced by a spring with a lower stiffness $k_g$ and the boundary point was set to 2.5 cm. This goal zone was followed by a blocking wall region with stiffness $k_w$ and boundary point of 6.5 cm. Consequently, the environmental forces can be described as:

1 $$\begin{aligned} \begin{aligned} F_{env} = 0&\text {, for } x_n < 0\\ F_{env} = k_t \cdot x_n&\text {, for } x_n \in [0.0, 2.5]\\ F_{env} = k_g \cdot (x_n - x_t)&\text {, for }x_n\in (2.5, 6.5]\\ F_{env} = k_w \cdot (x_n - x_t - x_g)&\text {, for } x_n > 6.5 \end{aligned} \end{aligned}$$

The hand manipulation forces were calculated per hand according to the hand position and velocity according to:

2 $$\begin{aligned} \begin{aligned} {F_{x,front} = (x_{front} - x_{n}) \cdot k_{n} + ({\dot{x}}_{front} - {\dot{x}}_n) \cdot d_{n}}\\ {F_{x,back} = (x_{back} - x_{n}) \cdot k_{n} + ({\dot{x}}_{back} - {\dot{x}}_n) \cdot d_{n}}\\ \end{aligned} \end{aligned}$$

where *x* and ${\dot{x}}$ are the position and velocity of the hand and needle object in x-direction, subscript $h = \{front, back\}$, refers to the front and back hand configuration present in the real world, subscript *n* refers to the needle object, and $k_n$ and $d_n$ are the spring and the damper connecting each hand to the needle object.

The environmental forces were combined with the hands’ forces to calculate the motion equation of the needle.

3 $$\begin{aligned} m_n \cdot \ddot{x}_{n} = (F_{front} + F_{back}) - F_{env} \end{aligned}$$

In this system, the direction of the hand manipulation forces was opposite that of the environmental force. To move the needle forward, participants had to produce a total positive force value between their hands that was higher than the environmental force. This can be done by both hands’ position leading the needle position or in the case where only one hand is leading the needle position and the other hand is lagging the needle position, the leading hand has to produce a positive force that will overcome both the environmental forces and the negative force generated by the lagging hand. Since the two hands were connected to the needle object at the same point, a position difference between hands compared to the initial distance led to one hand being pulled back (positive force, similar to the effect of the environmental spring) and the other hand being pushed forward (negative force, the hand is being pulled towards the leading hand). This could happen for any asynchrony in the movement of the hands. Solving Eq. 3 at each timestep during the trial we found the needle position. Based on this position we calculated the individual manipulation force for each hand and applied it on each of the participant’s hands via the haptic robots. Using this system, participants could experience forces originating from the needle position in the environment and indirectly also from the virtual spatial difference between the two hands. For example, if the hands were moving in a perfectly synchronous manner, the experienced force was only due to the environmental forces.

To reduce task complexity, movement was constrained to translation in the x-axis using a virtual channel. That is, we generated perpendicular forces based on the deviation from the axis according to:

4 $$\begin{aligned} \begin{aligned} F_{y,h} = -y_{h} \cdot k_{w} \\ F_{z,h} = -z_{h} \cdot k_{w} \end{aligned} \end{aligned}$$

where $k_w$ is the stiffness of the virtual wall and is identical to the blocking wall stiffness. Table 1 lists all environmental variables used for the simulation.

**Table 1:** Parameters of the virtual needle-tissue model and environmental variables

Variable	Abbreviation	Value			
Tissue stiffness	$k_{t}$ (N/m)	40	70	100	130
Goal stiffness	$k_{g}$ (N/m)	2			
Wall/Force channel stiffness	$k_{w}$ (N/m)	800			
Needle stiffness	$k_{n}$ (N/m)	200			
Needle damper	$d_{n}$ (Ns/m)	2			
Tissue thickness	$x_{t}$ (cm)	2.5			
Goal thickness	$x_{g}$ (cm)	4			
Needle mass	$m_n$ (kg)	0.010			

### Experimental paradigm

Each trial started with the needle outside of the virtual tissue. At the sound of an auditory cue, participants had to retract the needle by 2 cm and then push forward through the tissue into the goal region. Participants were asked to push into the tissue until they felt the breaking point, and then stop as quickly and as close as possible to the breaking point. Trials ended when the needle penetrated the tissue and the speed of the needle was below 0.1cm/s for 0.3s. All environmental forces were removed after trial completion. Participants received a score of 0 to 100, depending on the maximum insertion depth. From 100 points, 10 points were deducted per 0.4 cm penetration into the goal region with the score calculated as $score = \dfrac{x_{max} - x_t}{x_g} \cdot 100$, where $x_t$ is the thickness of the tissue and $x_g$ is the thickness of the goal zone. The experiment started with a familiarization stage in which participants performed 15 trials without forces (i.e. $k_t$, $k_g$, $k_w$ = 0) and 15 trials interacting with a tissue that had a stiffness value equal to the mean stiffness of all tissues presented in the experiment ($k_t$=85N/m). Following the familiarization stage, participants performed blocks of 15 trials in which they interacted with one of the four tissue stiffness values each (see Table 1). The blocked design allowed participants to adapt their strategy to a single stiffness value. This procedure was performed twice, once with the right hand in front (RiF) and once with the left hand in front (LiF). The tissue stiffness conditions and hand position order were pseudo-randomized between participants. An example of such an order is depicted in Fig. 1d.

### Data analysis

We performed data analysis and statistics in Python (version 3.11.4). Grip forces, as measured by the force transducers, were sampled at 500 Hz and filtered with a 20 Hz lowpass filter (zero-phase 6th order Butterworth). Kinematic and dynamic data produced by both hands were recorded at 1000 Hz and down-sampled to 500 Hz to match the grip force data. Our analysis focused on the kinematics and dynamics during interaction with the tissue, that is, during movement while compressing the spring representing the tissue and during the phase in which participants stopped their movement within the goal area. For the phase in which participants interacted with the tissue, we analyzed the interaction based on spatial information. That is, we examined participants’ movement, manipulation forces, or grip forces as a function of the insertion distance into the tissue. Since we did not force participants to move at a specific velocity or to complete the task in a specific time window, we resampled the data using linear interpolation to one sample per 0.01 cm so to compare trials of different insertion durations. This allowed us to compare Manipulation Force (MF) and Grip Force (GF) based on the Tissue Force (TF) at any given point during tissue interaction. Additionally, a separate analysis of GF around the time of tissue puncture was performed in the time domain.

#### Manipulation force

MF describes the force that was applied to the needle originating from the movement of one hand. We can calculate the manipulation force at any given point by plugging the transient hand position and velocity into the equation describing the spring-damper system connecting the hand and the needle (see Eq. 2, Fig. 1c). We excluded one participant from the analysis because the simulated forces exceeded the maximum force the robots could produce.

To analyze the contribution of each hand to the total force opposing the virtual tissue, after resampling data to the position-domain, we calculated the fraction of force applied by the hand of the total force applied by both hands, resulting in the fraction of contribution $C_{hand}(nu)$, by:

5 $$\begin{aligned} \begin{aligned} C_{front}(nu) = \dfrac{MF_{back}(x)}{MF_{front}(x) + MF_{back}(x)} \cdot 100\\ C_{back}(nu) = \dfrac{MF_{back}(x)}{MF_{front}(x) + MF_{back}(x)} \cdot 100 \end{aligned} \end{aligned}$$

In case the interaction forces between the two hands were larger than the environmental forces, e.g. if the physical distance between the hands was changed from the initial value, $C_{hand}$ could be negative or larger than 100. This was the case in some trials of the lowest $k_t$ and at the beginning of the tissue interaction. Points where $C_{hand}$ was outside of [$-$ 100, 200] were excluded, as they indicate that forces between the hands compressing or stretching the needle object outweighed those generated by the virtual tissue. Further, we rejected all trials with fewer than 50 samples within this region. This was equivalent to 0.9% of all trials.

To analyze the effort distribution between hands just before insertion, we performed an exponential regression on $C_{front}$ and $C_{back}$ in each trial. The convergence value of this regression was used to calculate the difference between contributions by $\Delta _{contribution} = C_{front} - C_{back}$.

For every participant, these $\Delta _{contribution}$ values were then averaged across trials for each hand configuration (RiF, LiF) and each $k_t$, resulting in eight values or four 2d data points. These points allow us to define the applied strategy as one of: right dominant, left dominant, front dominant or back dominant. For example, the right dominant strategy indicates that the right hand contributed a larger fraction than the left hand to the overall generated force (LiF: $C_{back} > C_{front}$; RiF: $C_{front} > C_{back}$) across both hand configurations (LiF, RiF). The back dominant strategy indicates that the back hand contributed a larger fraction to the overall forces than the back hand (LiF and RiF: $C_{back} > C_{front}$).

#### Grip force

GF describes the grasping forces between the index finger and thumb, as measured by the force transducers. This force was perpendicular to the MF. We excluded three participants from the grip force analysis whose total grip force (sum of left and right hand) was lower than the TF for more than 500 data points; this could happen if force transducers/robot end-effector were grasped in the wrong position.

***Insertion Phase*** As we expected a relationship between grip forces and manipulation forces applied by each hand, we performed a linear regression between GF as the dependent variable and MF as the independent variable in each trial, to analyze how GF was scaled according to the forces generated by the hand. In this analysis, we focused on the GF-MF trajectory when MF was positive. That is, we calculated the regression during interaction with the tissue where both hands were leading the needle position. This was usually the case when environmental forces were elevated. The slope of the resulting regression line was used for further analysis. Analogous to the manipulation force analysis, we defined $\Delta _{slope} = slope_{front} - slope_{back}$. We rejected all trials with fewer than 50 samples within the region of interest, resulting in 2.9% of trials being rejected for the analysis of GF scaling.

***Preparation and Reaction to Tissue Breaking Point*** We evaluated preparation and reaction to the tissue breaking point by analysing the grip force and grip force rate traces. For both configurations and hands, we ran a peak detection in the Grip Force Rate (GFR) before ($-$ 200ms$\le$t<25ms) and after (0ms<t$\le$150ms) the time of penetration, as these ranges have been reported as relevant for anticipatory [33–36] and reactive [33, 34, 36, 37] grip force responses in collisions. Based on the time of the anticipatory and the reactive $GFR_{max}$, we then found the time of the onset and maximum GF, by searching for the points where the GFR crossed 10% of the detected peak height.

Further, we determined the amplitude of each $GFR_{max}$ peak and the change in GF amplitude ($GF_{diff} = GF_{max}- GF_{onset}$) around each peak. If peak detection or 10% level detection failed, we excluded the corresponding peak resulting in the exclusion of 3.6% of all peaks.

#### Other movement kinematics

We analyzed different movement kinematic metrics to determine whether differences may influence manipulation and grip force control. For each trial, we calculated the following parameters:

***Completion Time*** In each trial, we recorded the duration *T* from the auditory cue at the beginning to the time when the needle object was held stationary in the goal zone.

***Max. Penetration Distance*** In each trial, we recorded the maximum penetration distance of the needle object into the goal zone ($x_{max}$) in the X direction before the trial was terminated.

***Movement Speed*** Using the recorded hand position (see Fig. 1), we calculated the movement speed in 3d, which was dominated by the X component, at each time point and calculated the mean value ${\bar{s}}_{hand}$ for each trial and hand.

***End-Effector Rotation*** While we asked participants to hold the thimbles in the same orientation throughout a trial, we recorded the orientation of the robot end-effector (hand) at each time point. Using Euler angles (yaw $\gamma$, pitch $\beta$, roll $\alpha$), we calculated the mean for each trial, angle, and hand.

#### Statistical analysis

Before statistical testing, we ran the Anderson-Darling normality test to determine whether or not the variables were normally distributed. To test the effect of hand configuration and environmental forces ($k_t$), on movement kinematics, we performed the Friedman test for repeated samples (factor = $k_t$, $k_t$ $*$ config.) and the Wilcoxon signed-rank test (factor = config.).

We then calculated the Spearman’s correlation between each kinematic metric and $\Delta _{slope}$ and $\Delta _{contribution}$, respectively, to rule out the possibility of a specific behaviour affecting our outcome measures. Statistical significance was determined at p <0.05 in all tests.

## Results

**Fig. 2:**
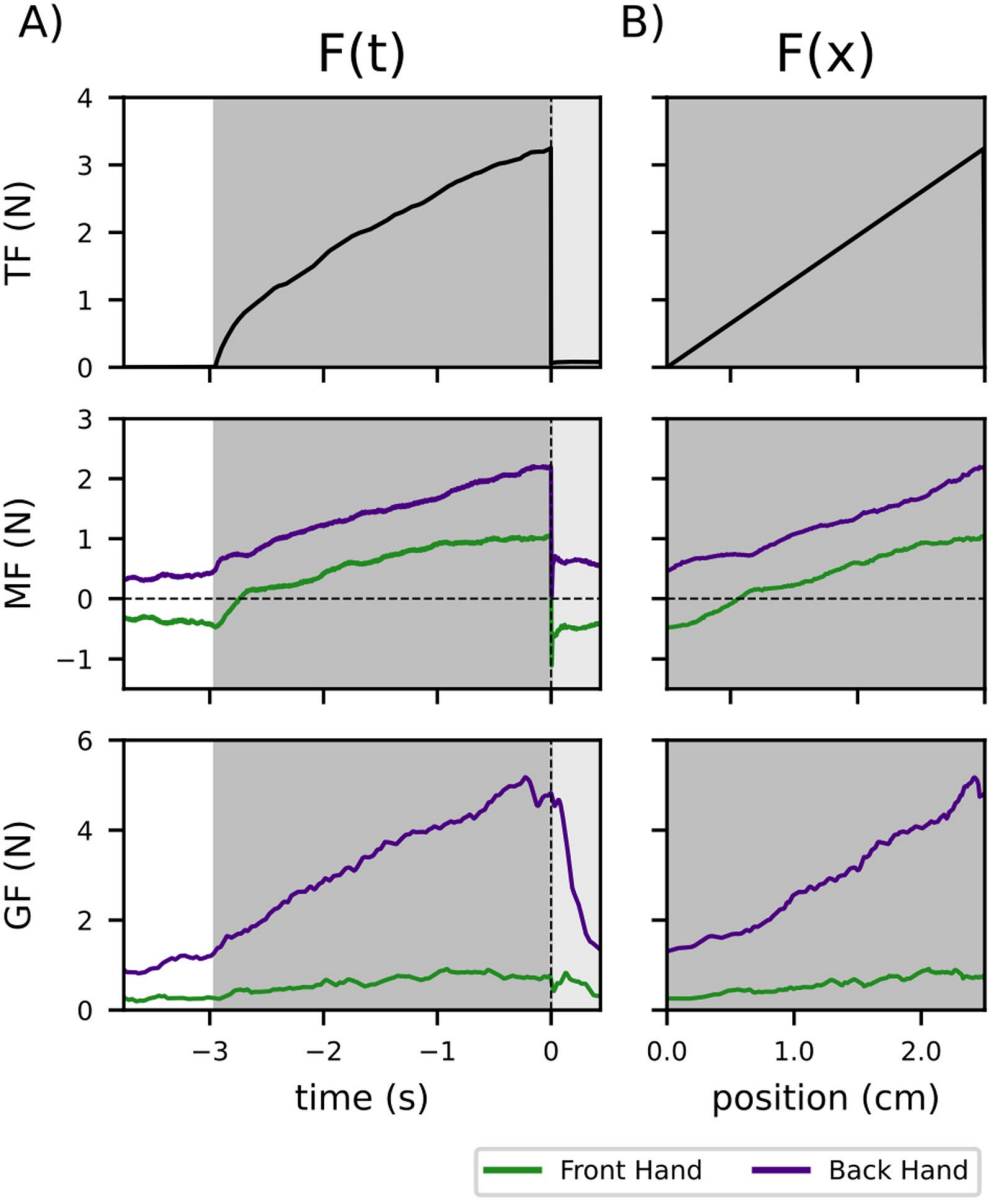
Example of manipulation and grip forces. **a** Examples of tissue, manipulation, and grip forces for the front (here: left) hand (green) and back (here: right) hand (purple) in the time domain for a single trial. **b** Same forces as in (A) as a function of needle position

Participants bimanually inserted a needle object into a simulated tissue in two different hand configurations. We measured manipulation force and grip force in both hands during the interaction with the virtual tissue and at the time of tissue puncture. Here, manipulation force refers to the force generated by each hand acting on the simulated needle. Examples of the force elevation and drop following insertion for both hands in the time- and position domains are depicted in Fig. 2.

### Learning effect

**Fig. 3:**
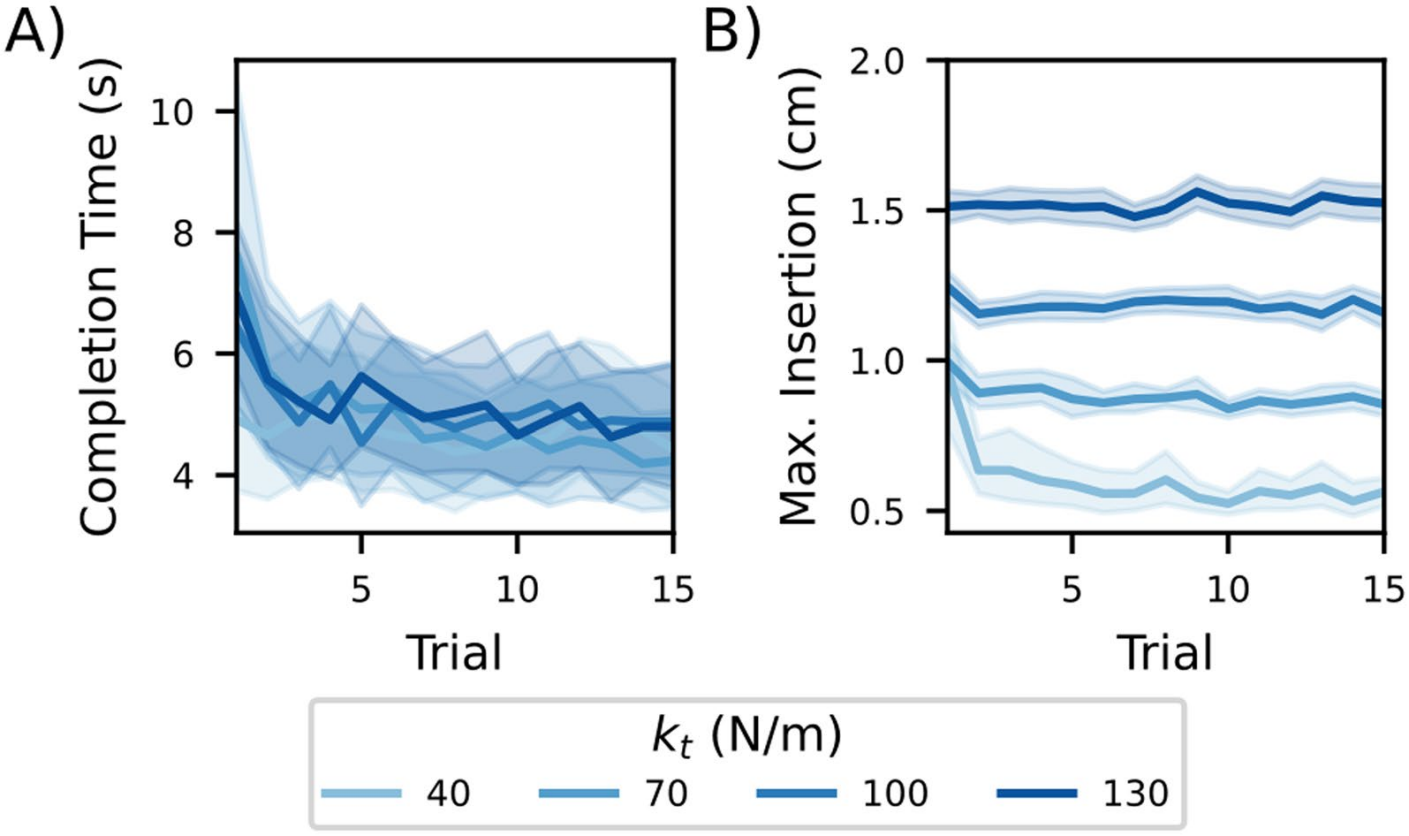
Learning effect across trials. **a** Completion time for individual trials reduced for each $k_t$ and converged to the same value (4.4±0.1s, mean and 95% confidence interval across trials 5–15). **b** Average maximum insertion and 95% confidence interval across trials for different $k_t$. Adaptation was prolonged for the lowest tissue stiffness (40N/m), but converged to steady values from trial 5

In each trial, participants were instructed to stop the needle and, therefore, the hand movement, immediately after the puncture of the tissue. Each participant completed four blocks of 15 trials each, in which one of the four possible stiffness values was used to simulate the tissue ($k_t$). To examine adaptation to the stiffness value within each block, we determined the completion time and maximum insertion distance into the goal zone for each trial. Participants’ completion time decreased over the first four trials and remained at a similar level regardless of $k_t$ and hand configuration (see Fig. 3a). Similarly, maximum insertion into the goal zone decreased over initial trials and then remained stable (see Fig. 3b). In the lowest tissue stiffness (i.e. $k_t$ = 40N/m), participants required the first five trials to reach a steady maximum penetration level; otherwise, we generally observed little to no performance improvement after the second trial. In both hand configurations, the maximum penetration distance scaled linearly with $k_t$. Most trials ended within the goal region, and only few trials across all participants ended with touching the blocking wall (0.2% of all trials).

### Manipulation force

**Fig. 4:**
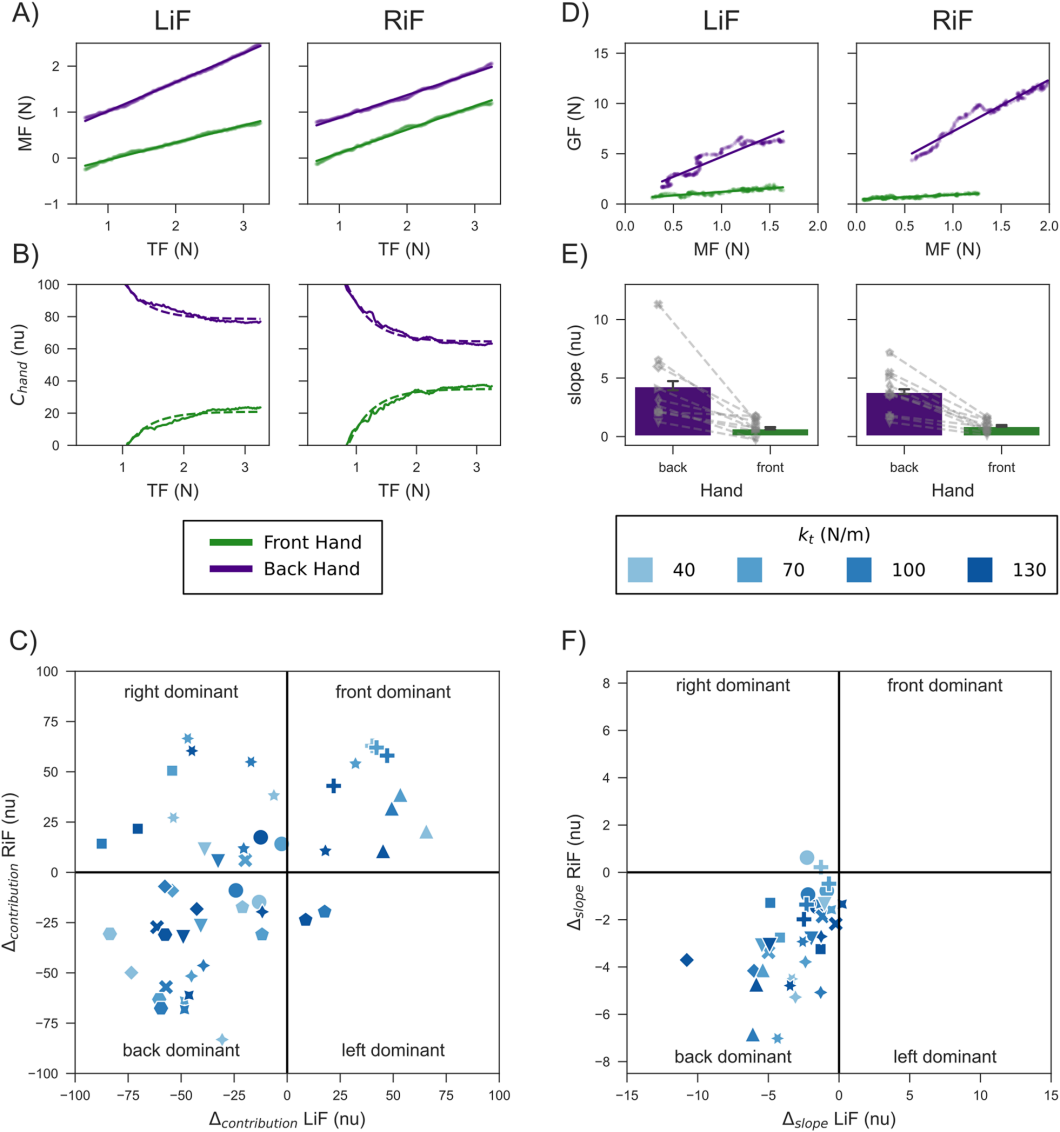
Analysis of MF and GF during tissue interaction. **a** Exemplary trial of the relationship between TF and MF for front (green) and back (purple) hand with linear regression lines. **b** Same example trial as in (**a**) showing the relationship between TF and fraction of contribution. The dashed lines depict exponential regressions that converge towards the final hand contributions ($C_{front}, C_{back}$) at tissue penetration. **c** $\Delta _{contribution}$ ($C_{front} - C_{back}$) between the two configurations with different shapes of markers for each individual. Each Quadrant resembles a different strategy for MF distribution between hands. **d** Exemplary trial of the relationship between TF and GF for front and back hand with linear regression lines. **e** The mean slope from (**d**) between hand positions with errorbars indicating 95% confidence interval. Gray markers depict the mean for individual participants. **f** $\Delta _{slope}$ ($slope_{front} - slope_{back}$) between the two configurations with different shapes of markers for each individual. Each Quadrant resembles a different strategy for GF scaling distribution between hands

During tissue interaction, the manipulation forces of both hands scaled linearly with the tissue force (examples of trials for one participant in the two hand configurations in Fig. 4a). When transforming the same data to fraction of contribution ($C_{hand}(nu)$), we observed an exponential convergence in both the front (green) and back (purple) hands (Fig. 4b). In this example, the participant preferred the back hand as the main force contributor during the interaction, regardless of the hand configuration. To examine participants’ strategies, we calculated for each participant the $\Delta _{contribution}$ for each experimental hand configuration (LiF and RiF) which set four unique strategies based on the sign of the $\Delta$ (see Fig. 4c). Each quadrant represents a specific strategy, with the example participant appearing in the 3rd quadrant indicating the back dominant strategy. We repeated this procedure for each participant and for each tissue stiffness value (four in total). In most cases, participants applied the back dominant or the right dominant strategies, while the front dominant and left dominant strategies were used less frequently. Overall, the strategy of the applied manipulation forces varied between participants and between tissue stiffness values. While we did investigate movement in the non-task relevant plane (YZ-plane), the strategies we observed did not correlate with those visible in the task-relevant plane.

### Grip force

***Insertion Phase*** Examining the grip force and manipulation force (GF-MF) relationship, we observed a clear scaling of the GF in both hands (see an example in Fig. 4d). The GF increased as MF increased for both hands, but not at the same rate. To analyze this difference, we fitted a linear regression to the trajectories of the front and back hands in the GF-MF plane and extracted the slope of this regression line (see Fig. 4d). The slope revealed a clear difference between the front and back hands. In both hand configurations, the $GF_{front}$ scaled proportionally to MF with a slope between 0 and 1, while $GF_{back}$ showed on average an increase in GF of 4[N] for every 1[N] increase in MF (see Fig. 4e).

When averaging across the four stiffness values, we observed a similar trend across all participants. They all exhibited higher GF scaling for their back hand compared to the front hand, although the scaling value differed between participants (see Fig. 4e). To further understand the nature of applied GF, we analysed the behavior for each stiffness value similarly to the analysis of the manipulation forces. That is, we calculated the difference in slope value between the front hand and back hand ($\Delta _{slope}$) for each hand configuration (LiF and RiF) and each participant, which again set four unique strategies based on the sign of the $\Delta$ (Fig. 4f). With two exceptions in lower spring constants, all participants demonstrated the back dominant strategy, meaning the slope between GF and MF was higher in their back hand regardless of hand configuration. This was independent of the MF employed by the participants. Even participants who used the right or front dominant strategies for the MF showed a lower slope in the front hand compared to the back hand. Overall, this suggests that participants adapt the GF-MF scaling in the insertion phase according to hand position within the hands’ configuration, not to the hand leading the movement in terms of contribution effort.

***Preparation and response to Tissue Puncture***

**Fig. 5:**
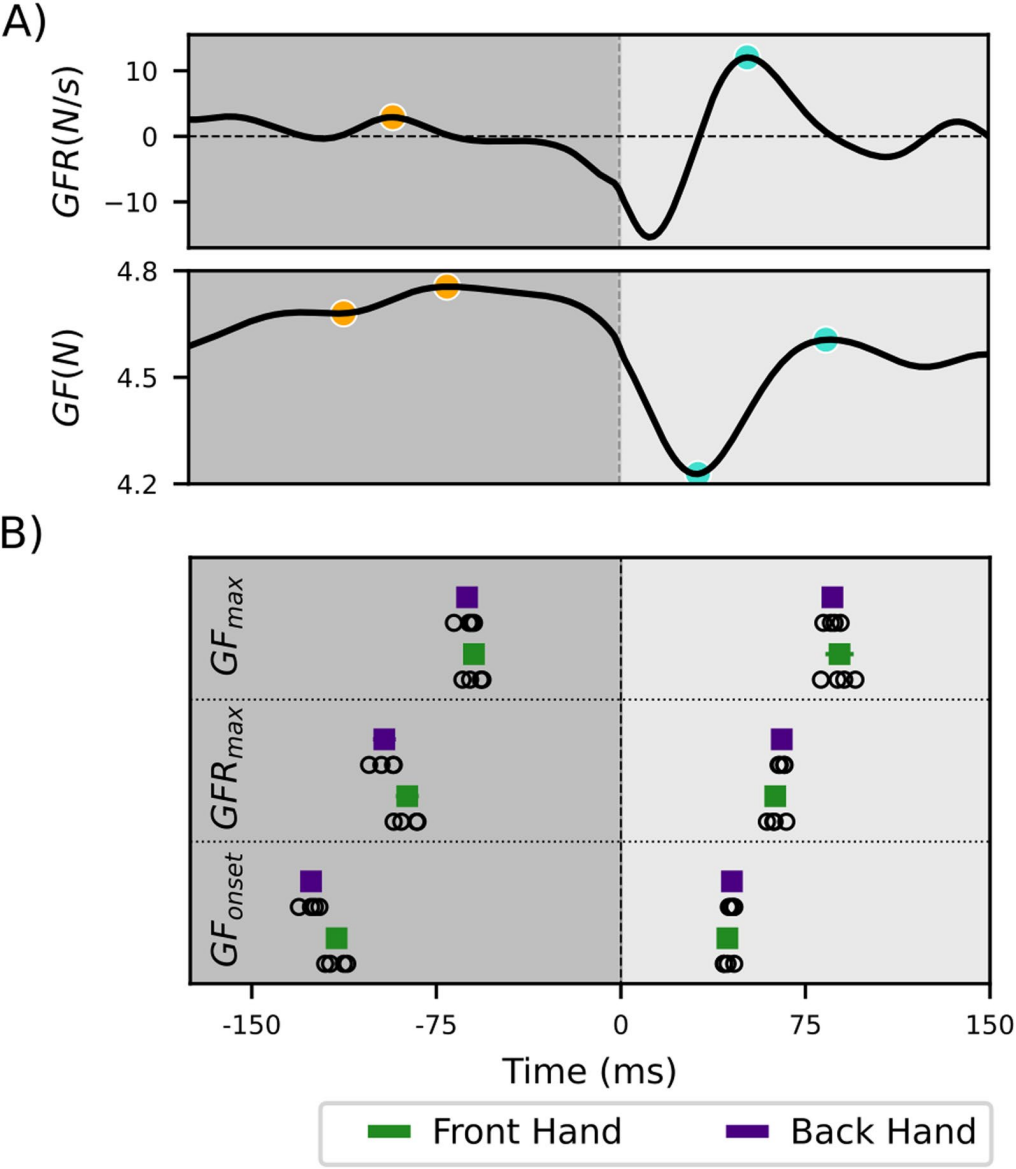
Timing of preparatory and reactive grip force changes around time of tissue puncture. **a** Exemplary trial with detected $GF_{onset}$, $GF_{max}$, $GFR_{max}$ before (orange) and after (turquoise) puncture. **b** Pooled time of $GF_{onset}$, $GF_{max}$, $GFR_{max}$ before and after penetration for front (green) and back (purple) hands. Square markers indicate the mean, whereas error bars indicate the 95% confidence interval across $k_t$. Note that these confidence intervals are small such that they are mainly contained within the markers. Gray circular markers show the mean value across participants and hands per $k_t$

Anticipatory and reactive grip forces around the puncture time point of the needle were analyzed in the time-domain. In a typical trial, we observed two peaks in the GF rate (GFR) trace. An anticipatory increase before the puncture, followed by a local minimum and a steep reactive increase (see example traces Fig. 5a). The detected anticipatory $GFR_{max}$ occurred slightly earlier in the front than the back hand (Table 2, Fig. 5b). A repeated measures ANOVA showed a significant effect of $k_t$ (F(3,27)= 3.443, p = 0.031), but not the hand position (front, back; F(1,9)=3.367, p = 0.100) and the hand (left, right; F(1,9)=0.650, p = 0.441) on the time of anticipatory grip force rate peak. Post-hoc paired ttest with Bonferroni correction revealed no significant effect of $k_t$. The corresponding detected anticipatory $GF_{onset}$ and $GF_{max}$ showed the same pattern. Compared to the anticipatory peak, the reactive response was slightly less variable across participants and hands (Table 2, Fig. 5b). In general, we observed a slightly higher variability in the front compared to the back hand in both increases and in the anticipatory compared to the reactive increase. However, there was no significant effect of hand, hand configuration or tissue stiffness on the timing of grip force responses.

**Table 2:** Detected time of grip force onset, grip force rate maximum and grip force maximum in relationship to the tissue breaking point in ms

Variable	Hand position	Anticipatory		Reactive	
		Mean	Ci	Mean	Ci
$GF_{onset}$	Front	$-$ 116	4.2	43	1.9
$GF_{onset}$	Back	$-$ 126	3.5	45	1.0
$GF_{max}$	Front	$-$ 60	3.8	89	5.7
$GF_{max}$	Back	$-$ 63	3.6	86	2.9
$GFR_{max}$	Front	$-$ 87	4.7	63	3.2
$GFR_{max}$	Back	$-$ 96	4.6	65	1.2

Data are pooled for each $k_t$, participant and trials 5–15. Confidence interval is shown across $k_t$

**Fig. 6:**
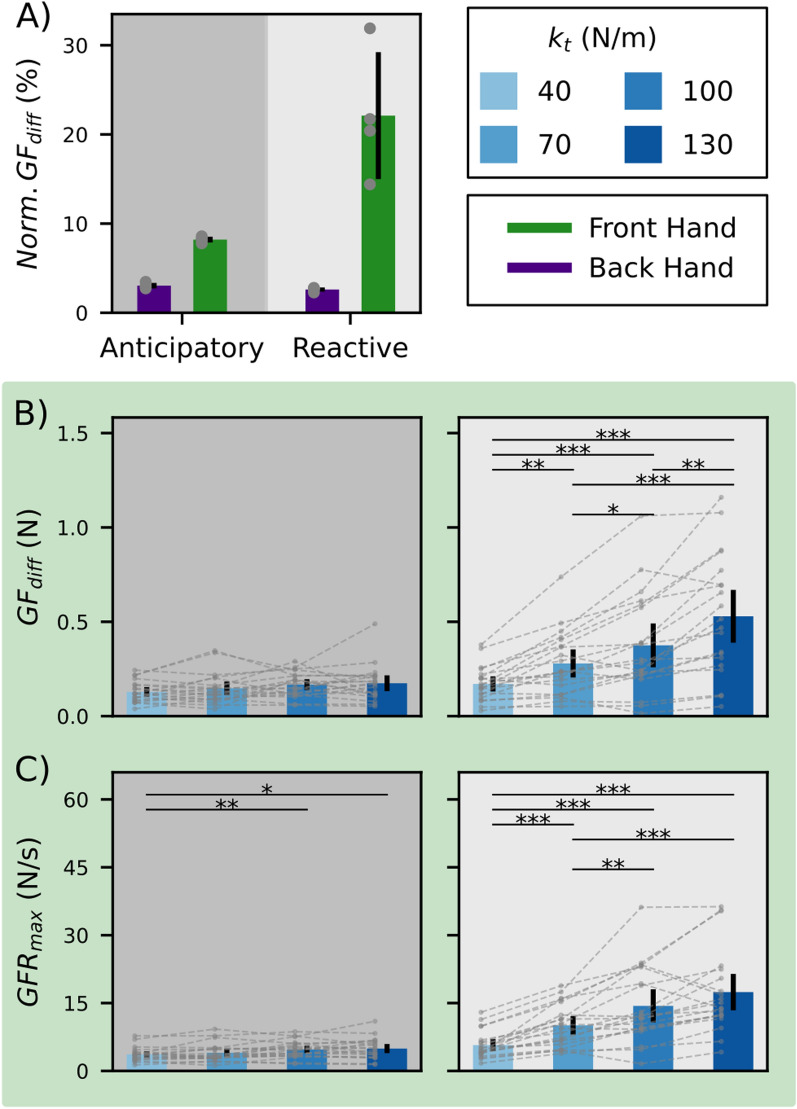
Grip force and grip force rate amplitudes of detected peaks before and after tissue puncture. **a** Normalized $$GF_{diff}$$ (to *GF* at time of insertion) in front and back hands for anticipatory and reactive peaks. Gray circular markers show the mean value across participants and hands per $$k_t$$, error bars the 95% confidence interval across $$k_t$$. **b** Amplitudes of $$GF_{diff}$$ in the front hand for anticipatory and reactive peaks. Bars indicate the 95% confidence interval. **c** Amplitudes of $$GFR_{max}$$ in the front hand for anticipatory and reactive peaks. Bars indicate the 95% confidence interval. Horizontal lines show statistically significant differences between conditions, with $$*$$-p < 0.05, $$**$$-p < 0.01, $$***$$-p < 0.001

Using the described time points, we investigated the amplitudes of GF and GFR signals before and after the puncture. As reported above, the grip forces in the back hand increased at a higher rate throughout the movement inside the simulated tissue, which set an elevated baseline value for the back hand compared with the front hand at the time of the puncture. For this reason, when comparing the amplitude of the anticipatory and reactive grip force peaks to the GF baseline of each hand (GF at the time of puncture), we observed that only the peaks of the front hand were significant to the general GF signals (Fig. 6a). Further analysis of the anticipatory and reactive peaks for the front hand showed that tissue stiffness $k_t$ affected the peak amplitude. We observed a significant increase in peak amplitude for the anticipatory peak when increasing the $k_t$ (RM ANOVA: F(3,27)=5.734, p = 0.003, Fig. 6b, left panel), however, post-hoc paired ttests with Bonferroni correction revealed no significant effect of $k_t$. For the reactive peak, we observed a clear amplitude scaling to the $k_t$ value (RM ANOVA: F(3,27) = 6.151, p = 0.003, Fig. 6b, right panel). Post-hoc ttests with Bonferroni correction revealed significant differences between different values of $k_t$ (40–70:t(19) = $-$ 4.616, p = 0.0011; 40–100:t(19)= $-$ 4.757, p = 0.0008; 40–130:t(19) = $-$ 6.280, p < 0.0001; 70–100:t(19) = $-$ 3.584, p = 0.0119; 70–130:t(19) = $-$ 5.275, p = 0.0003; 100–130:t(19) = $-$ 4.470, p = 0.0016). A similar trend was evident for the $GFR_{max}$ (Fig. 6c, right panel), suggesting that the shape of reactive response was also scaled to the tissue stiffness with sharper GF increase when increasing the $k_t$ (RM ANOVA: F(3,27) = 44.280, p < 0.0001; 40–70:t(19) = $-$ 6.948, p < 0.0001; 40–100:t(19) = $-$ 6.286, p < 0.0001; 40–130:t(19) = $-$ 7.270, p < 0.0001; 70–100:t(19) = $-$ 3.838, p = 0.0067; 70–130:t(19) = $-$ 4.906, p = 0.0006; 100–130:t(19) = $-$ 2.673, p = 0.0901). Analysis of the anticipatory GFR also revealed significant differences (RM ANOVA: F(3,27) = 5.734, p = 0.0036; 40–70:t(19) = $-$ 1.250, p = 1.3597; 40–100:t(19) = $-$ 3.741, p = 0.0083; 40–130:t(19) = $-$ 3.036, p = 0.0408; 70–100:t(19) = $-$ 2.352, p = 0.1778; 70–130:t(19) = $-$ 2.020, p = 0.3462; 100–130:t(19) = $-$ 0.623, p = 3.2457). The analysis of the peak modulation according to $k_t$ for the back hand showed similar results: higher modulation of the reactive peak than the anticipatory peak. However, the overall amplitudes were low compared to the front hand and had a reduced effect on the overall GF signal.

To summarize, reactive GF responses in the front hand, regardless of left or right, were modulated with $k_t$ (Fig. 6b). The reactive GFR in the front hand also showed a clear modulation with $k_t$, while back hand and anticipatory $GFR_{max}$ did not show this clear pattern. When scaling the amplitudes of $GF_{diff}$ and GFR to the applied grip force at insertion, it is evident that the front hand responses show higher relative modulation.

### Other movement kinematics

We tested for effects of participant, hand configuration and $k_t$ on maximum penetration, trial duration and a number of movement kinematics (see Table 3). As expected, the maximum penetration was affected by $k_t$. Further we could see an effect of configuration on the pitch of the left hand (${\bar{\beta }}_{l}$) and the yaw of both hands (${\bar{\gamma }}_{l}$, ${\bar{\gamma }}_{r}$). Contrasting clear patterns in grip force control across $k_t$ and hand configurations, we could not observe an effect of movement kinematics on these measures. We also evaluated the correlation between $\Delta _{contribution}$ and movement kinematics as well as $\Delta _{slope}$ (see Table 4). The only statistically significant correlation was the $\Delta _{contribution}$ to the yaw of the left hand (${\bar{\gamma }}_{l}$), which we deem as irrelevant as the correlation is <0.5. In summary, we did not observe any relevant interaction between movement kinematics in different conditions. Therefore, the observed differences in manipulation and grip forces cannot be explained by differences in movement kinematics.

**Table 3:** Test statistics and *p*-values for outcome variables and movement kinematics

Dependent variable	$x_{max}$		*T*		${\bar{s}}_{r}$		${\bar{s}}_{l}$		${\bar{\alpha }}_{r}$	
Factor/interaction	$\chi ^2$/W	P	$\chi ^2$/W	P	$\chi ^2$/W	P	$\chi ^2$/W	P	$\chi ^2$/W	P
$k_t$	30.000	<**0**.**001**	2.760	0.430	0.360	0.948	0.240	0.971	1.560	0.668
Configuration	22.000	0.625	21.000	0.557	18.000	0.375	11.000	0.105	16.000	0.275
$k_t *$ configuration	66.900	<**0**.**001**	7.467	0.382	3.567	0.828	5.367	0.615	15.767	0.027

Dependent variable	${\bar{\alpha }}_{l}$		${\bar{\beta }}_{r}$		${\bar{\beta }}_{l}$		${\bar{\gamma }}_{r}$		${\bar{\gamma }}_{l}$	
Factor/interaction	$\chi ^2$/W	P	$\chi ^2$/W	P	$\chi ^2$/W	P	$\chi ^2$/W	P	$\chi ^2$/W	P
$k_t$	1.560	0.668	1.320	0.724	0.840	0.840	0.120	0.989	6.840	0.077
Configuration	16.000	0.275	8.000	0.049	2.000	**0**.**006**	7.000	0.037	0.000	**0**.**002**
$k_t *$ configuration	7.333	0.395	16.233	0.023	37.133	<**0**.**001**	18.667	**0**.**009**	49.000	<**0**.**001**

$x_{max}$-maximum insertion distance; *T*-Completion time; ${\bar{s}}_{l,r}$-Mean movement speed (left, right); ${\bar{\alpha }}_{l,r}$-end-effector roll (left, right); ${\bar{\beta }}_{l,r}$-end-effector pitch (left, right); ${\bar{\gamma }}_{l,r}$-end-effector yaw (left, right)

Bold values indicate significance after Bonferroni correction

**Table 4:** Correlations and *p*-values for outcome measures and movement kinematics

$$\Delta _{slope}$$										
Variable	$x_{max}$	*T*	${\bar{s}}_{r}$	${\bar{s}}_{l}$	${\bar{\alpha }}_{r}$	${\bar{\alpha }}_{l}$	${\bar{\beta }}_{r}$	${\bar{\beta }}_{l}$	${\bar{\gamma }}_{r}$	${\bar{\gamma }}_{l}$
Corr	$-$ 0.0068	0.0446	0.0198	0.0232	$-$ 0.1559	0.1649	$-$ 0.0677	0.1298	$-$ 0.0057	0.0868
p	0.9535	0.7004	0.8645	0.8414	0.1756	0.1519	0.5587	0.2606	0.9611	0.4528
$$\Delta _{contribution}$$										
Variable	$x_{max}$	*T*	${\bar{s}}_{r}$	${\bar{s}}_{l}$	${\bar{\alpha }}_{r}$	${\bar{\alpha }}_{l}$	${\bar{\beta }}_{r}$	${\bar{\beta }}_{l}$	${\bar{\gamma }}_{r}$	${\bar{\gamma }}_{l}$
Corr	$-$ 0.0528	$-$ 0.0243	$-$ 0.0211	$-$ 0.0161	0.2760	0.0441	0.1562	$-$ 0.2075	$-$ 0.1314	0.4444
p	0.6438	0.8315	0.8534	0.8881	0.0138	0.6995	0.1694	0.0666	0.2484	<**0**.**001**

$x_{max}$-maximum insertion distance; *T*-Completion time; ${\bar{s}}_{l,r}$-Mean movement speed (left, right); ${\bar{\alpha }}_{l,r}$-end-effector roll (left, right); ${\bar{\beta }}_{l,r}$-end-effector pitch (left, right); ${\bar{\gamma }}_{l,r}$-end-effector yaw (left, right)

Bold values indicate significance after Bonferroni correction

## Discussion

We examined the effect of hand configuration on grip and manipulation forces in a simulated bimanual needle insertion task. Using a coupled bimanual movement, where both hands are tasked with moving the object, we observed multiple strategies for generating manipulation force profiles across participants. In contrast, grip forces were scaled consistently during tissue interaction; the back-hand grip force always showed a higher grip force-manipulation force slope than the front hand. At the moment of tissue puncture, both the front and back hands exhibited stereotyped timing patterns in grip force, consisting of a double peak around the puncturing time. While the relative amplitude of preparatory increases in the front-hand was larger than in the back-hand, it did not scale to the environment stiffness ($k_t$) in either hand. The reactive grip force response also showed a significantly larger amplitude in the front than back-hand, but in contrast to the predictive response, it was also scaled with the environment stiffness.

Similar to previous work [18–20, 25], our results indicate that trajectory control (here represented by manipulation force) and grip force are modulated by distinct mechanisms. In the insertion phase, we observed a pattern in grip force control, characterized by an elevated grip force slope in the back compared to the front hand. In contrast, the effort distribution between hands showed a wide range of strategies, which seemed to be individual. Although grip force control and trajectory control are usually coupled [1–3, 14–16], previous work demonstrated similar dissociation between the two in unimanual [18–20] and bimanual [25] tasks. Our findings extend those results by showing the effect of physical hand configuration on manipulation forces but not on grip forces. One possible explanation for the difference between the control of these forces is the idea that grip force is more likely to be the outcome of an unconscious, implicit process [23], whereas the generated manipulation force required for this task resulted from explicit planning.

The degree to which each hand contributed to the overall force was mediated either by the hand configuration (front dominant and back dominant) or handedness (right dominant) (see Fig. 4c). While we observed some indication of a role distribution between hands for each individual, our data do not show a general strategy across participants. This lack of a unifying strategy in manipulation force generation may be the result of redundancy innate to our task. Previous work investigating role distribution between the hands focused on unimanual movements of both arms (e.g. [10]). Especially relevant to this study was the study of Gershon et al., who showed that hand dominance improves performance in an unimanual puncture task [38]. Other studies examined role distribution by imposing a stabilizer and actor roles onto the arms [9]. In the few experiments where participants were free to choose a strategy, it has been found that the dominant arm takes a stronger stabilizing role when both hands are tasked with stabilizing the same object [8]. While this was not the case in our study, as hand configuration rather than hand dominance influenced the manipulation forces of some of the participants, it is important to note that our study was limited to right-handed participants. Although previous studies also supported the idea that hand dominance did not affect motor performance, such as during grasping [39] or precision grip [40], extending our current results to left-handed individuals will be the subject of future work.

In contrast to our findings on MF, the analysis of GF during tissue interaction revealed one common strategy across all participants. All participants exhibited a higher GF scaling in the back hand regardless of hand configuration. Therefore, only the relative hand position (front, back), not the hand dominance (right), affected the GF pattern. Notably, this effect was independent of the MF strategy, i.e. even subjects who applied higher MF with the front hand showed a higher GF slope in the back hand. While there is little previous research on grip force scaling in bimanual tasks, grip force has been used to quantify prediction and implicit adaptation (e.g. [41]). The predictive quality of grip forces was evidenced e.g. by predictive scaling to load forces in point-to-point and cyclic movements [3, 15]. Although we could not observe a role distribution between the hands into leader-follower/actuator-stabilizer according to the MF, the pattern observed in the GF might indicate a role distribution according to hand position in a bimanual configuration. One explanation for this could be different controllers or cost functions for the hands depending on the position [42]. While it has previously been proposed that such a mechanism is mediated by hemispheric specialisation (e.g. [43]), our data indicates a more flexible control based on position and task requirements rather than hand dominance. An alternative explanation for different GF scaling could be that the back hand’s movement direction is more in line with its expected stiffness ellipse, which has been shown to result in higher grip forces [44, 45]. Although our results cannot differentiate between these explanations, it does suggest that hand position is an important control variable for bimanual grip force behaviour. Further experiments, including an additional configuration where the two hands are next to each other or a pulling rather than pushing task, could help to elucidate this question.

Investigating grip force patterns around the time of insertion, we found a replicable preparatory increase in GF regardless of hand and hand configuration. This is consistent with previous work investigating unimanual grip force in collisions. In highly predictable scenarios, the increase starts before the time of impact. It continues until after without intermittent plateauing around the time of impact, as reported in self-inflicted time-locked collisions [36, 46] and self-inflicted [16] and passive [35] position-locked collisions with visual information about the obstacle. In less predictable tasks similar to ours, a trough in GFR after the preparatory increase, resulting in a double peak of GF and GFR around the time of impact was observed [33, 47].

Similarly, we observed a reactive increase in GFR and, consquently, GF, right after the time of the puncture. The onset of this increase occurred within a mean of 44ms of the time of tissue penetration, with the maximum in GFR at a mean of 64ms and a maximum in *GF* at a mean of 87ms. The timing of $GF_{max}$ is in line with previously reported values in experiments where collisions were imposed on a hand-held object and participants received visual information about the time of collision [16, 33, 36, 47–49]. Johansson and Westling assumed supraspinally mediated prestructured motor commands to be responsible for the fast responses they observed. However, the onset of GF response appears earlier in our data than the 60–90ms described in other work [2, 36], therefore, long-latency reflexes can be excluded in our case. Corrective changes in grip force in response to slip can be as short as 50ms [50], therefore, we hypothesize that the observed short delay is caused by short-latency spinal reflexes triggered by movement of the arm and shoulder at the time of tissue puncture causing shorter conduction times and faster onset of GF response.

Previous studies reported a scaling of GF and GFR amplitudes both before [16, 33, 35] and after [2] collisions. Our results demonstrated a scaling to $k_t$ in reactive, but not anticipatory increases. The reactive response amplitudes scaled with $k_t$ and were much more prominent in the front hand compared to the back hand. This effect was evident in both hand configurations and was not affected by which hand was in front. We hypothesise that, due to higher scaling during the tissue interaction, the grip force in the back hand was already saturated, and the effects of the puncture were therefore amplified in the front hand.

Across each simulated tissue stiffness, we could observe learning as evidenced by a reduction in completion time and the maximum distance traveled into the goal zone. In comparable work, this learning was also visible in a time shift of the anticipatory [35, 36, 41] and reactive [48] responses. Our data did not show such an adaptation. A factor that might reduce the learning effect on these parameters in our experiment could be limited information about the time of impact. Participants had to estimate the point of tissue breakage based on the position of force and the force magnitude acting on their hands. This information is noisier than, for example, visual feedback of a ball perturbing a hand-held object [35], which can limit the ability to predict and adjust the temporal profile of the grip force. In experiments where limited information about the perturbation was available to participants [41, 48], this adaptation was evident only after 30–40 trials. As our participants completed only 15 trials per condition, it can be speculated that our trial number was too low to see the reported effect. We hypothesize that an increased number of repetitions might lead to a reduction of variability of the peak timing, however, a convergence to a single peak perfectly aligned with the tissue puncture is unlikely.

The bimanual coordination in force production, especially in grip force, presents a promising direction for rehabilitating neurological disorders such as rehabilitation after stroke. While most rehabilitation techniques target improving the motor performance of the affected arm, unilateral stroke usually also affects the performance of the ipsilesional arm [51]. Since most daily activities involve bimanual coordination, it was suggested that solely focusing on restoring the motor abilities of the affected arm is limited [52]. Instead, bimanual tasks may influence the weaker arm more since motor control centers of the unaffected arm may control the movement of both arms during joint limb movements, triggering more information transfer to and from the affected centers [53]. Our results suggest that using bimanual tasks, we can target either arms to take a bigger role in grip and manipulation force production based on the spatial configuration. However, since we did not test stroke patients, additional research is required to expand these results to this group. This can also be done using real objects instead of the virtual springs we used here in cases where patients cannot interact with virtual objects [54]. Although the extent to which the observed strategies generalize to real viscoelastic objects and to scenarios in which movement is not constrained to a single dimension remains to be determined [55], the force-based task and the idea of separated control mechanisms for grip and manipulation force is a promising direction in neurorehabilitation.

## Conclusion

We demonstrated that while grip force strategies were consistent across participants and configurations, showing elevated magnitude in the back hand compared to the front hand, there was no clear pattern in the generated manipulation forces. We propose that this separation occurred due to distinct mechanisms for manipulation and grip forces in our experiment. One reason for this separation could be explicit strategies affecting manipulation forces and implicit adaptation affecting grip forces.

Although there is no evidence for a role distribution between hands in MF data, we show that a preference for stabilizing and actuating roles is evident in the grip force data. These roles were not mediated by hand dominance but by hand position (front, back), which is in line with a flexible change of roles in bimanual manipulation according to task requirements. Compared to real-world object manipulation the presented task is simplified; the extent to which the observed strategies generalize to more complex manipulation remains to be determined.

## Data Availability

The data used in this study may be made available by the corresponding author upon a request.
